# Occupational interventions and their effects on work-related and mental health outcomes: A scoping review protocol

**DOI:** 10.1371/journal.pone.0354462

**Published:** 2026-07-24

**Authors:** Behdin Nowrouzi-Kia, Hong Ki Chloe Lau, Sana Siddiqui, Raihana Premji, Grant Hamblin, Armaan Rehman Shah, R. Nicholas Carleton

**Affiliations:** 1 Department of Occupational Science and Occupational Therapy, Temerty Faculty of Medicine, University of Toronto, 500 University Avenue, Toronto, Ontario, Canada; 2 Krembil Research Institute-University Health Network, Toronto, Ontario, Canada; 3 Institute for Mental Health Policy Research, Center for Addiction and Mental Health, Toronto, Ontario, Canada; 4 Department of Psychology, University of Regina, Regina, Canada; University of Foggia: Universita degli Studi di Foggia, ITALY

## Abstract

**Background:**

The well-being of individuals within their workplace (i.e., workplace mental health) has important implications for their productivity and mental health. The extensive literature on the effectiveness of individual workplace interventions does not yet include a comprehensive synthesis of occupational interventions delivered in workplace settings.

**Objective:**

The proposed scoping review is designed to identify and categorize occupational interventions studied in the literature; and 2) map reported effects on work-related outcomes and mental health outcomes.

**Methods:**

The review will examine five online databases to identify relevant articles: Embase, CINAHL, APA PsycINFO, MEDLINE, and Web of Science. The inclusion criteria will encompass peer-reviewed English-language studies published from 2005 to 2025, focusing on the effects of occupational interventions designed to improve workplace mental health. The results will support work participation among working adults.

**Discussion:**

The review will examine the effects of current occupational interventions on workers’ mental health and well-being, as well as on work-related outcomes (e.g., work participation, productivity). The review results can inform researchers designing future studies assessing the effectiveness of occupational interventions on mental health and guide the development of new interventions.

## Introduction

Mental health disorders are widespread among working adults, with 15% of the working-age adult population reporting having a mental disorder in 2019 [1]. Workplace mental health refers to the emotional, functional, psychological, and social well-being of individuals within their work environment. Workplace mental health encompasses how employees function and interact at work, including their ability to manage stress, remain productive, and maintain positive relationships [2]. Poor mental health is now globally recognized as one of the leading causes of productivity loss, burnout, and early workforce exit. Stress, depression, and anxiety account for an estimated 12 billion lost workdays annually, costing the global economy $1 trillion USD each year in productivity loss [3]. Mentally healthy workplaces are associated with higher engagement, satisfaction, and retention rates [4]. Most of the global adult population participates in the workforce, underscoring workplace mental health as a public health and occupational priority.

Workplace mental health and work outcomes are tightly interdependent: mental ill-health reduces attendance and on-the-job functioning, and is exacerbated by adverse psychosocial working conditions [5]. The proposed scoping review defines relevant work outcomes as absenteeism, presenteeism, productivity/workability, job satisfaction, and retention/return-to-work. In relation to work, absenteeism refers to being absent from work, whereas presenteeism refers to being present at work but functionally absent [6]. Productivity in the context of work can be understood as an employee’s ability to complete a given task in an efficient (i.e., completed within a given time) and effective manner [7]. Job satisfaction refers to a person’s subjective evaluation regarding their work experiences through feelings of contentment, fulfillment. and happiness [8]. Return to work refers to the resumption of occupational duties/work after a prolonged period of absence [9]. Poor mental health is consistently associated with higher sick-leave, diminished productivity, and increased turnover; concurrently, supportive work participation and occupation-focused supports (e.g., structured return-to-work programs, job-redesign, occupation-based rehabilitation) can facilitate recovery and improve work ability. Supportive work participation and occupation-focused supports produce variable impacts and effect sizes across intervention types and sectors. [10]. The variability can be partially explained by the inconsistent relationship between symptom reduction and restored occupational functioning [11,12]. The proposed review will extract and map both validated mental-health measures and the work-related outcomes listed above to assess intervention impact across clinical and real-world functional domains.

Occupational therapy supports quality of life and promotes health and well-being by enabling clients to participate in their desired and necessary daily occupations [13]. There are a wide variety of occupational therapy interventions, including activities, education and training, group and virtual sessions, and strategies that foster engagement in specific occupations [14]. The extant occupational therapy interventions can substantively improve mental health outcomes. According to the American Occupational Therapy Association (AOTA), occupational therapy practitioners are qualified to help individuals engage meaningfully in daily life [15]. Occupational therapy interventions can help individuals experiencing mental health challenges integrate into their communities, assume new roles, and (re)establish daily routines. Many workplace mental health interventions can reduce anxiety and depression at work [16]. Previous systematic reviews have evidenced occupational therapy interventions can positively influence work outcomes, including improved employee productivity and work ability, and can decrease absenteeism [10,17]. Occupational therapy interventions play an essential role in improving mental health and related outcomes within the workforce.

While occupational therapy interventions may influence work outcomes, they represent interventions from a single discipline. To allow for a greater scope, we will operationalize occupational interventions to include any intervention that directly targets work participation, functioning, and work-related mental health outcomes, regardless of the discipline. For example, this may include workplace mental health interventions, physical activity interventions, or vocational rehabilitation interventions [18–20].

There is extensive literature on the effectiveness of individual workplace interventions on work-related or mental health outcomes. However, there are limited comprehensive syntheses that examine multiple occupational interventions, across the general working population, and map their efficacy on work-related and mental health-related outcomes in a single review. The proposed scoping review will examine which types of occupational interventions have the most robust evidence, how the same interventions may differentially impact work outcomes and mental health outcomes, and describe evidence of variations in effectiveness across sectors. The proposed study will map and synthesize evidence on occupational interventions that support workplace mental health. Specifically, we will: 1) identify and categorize occupational interventions studied in the literature; and, 2) map reported effects on work-related outcomes and mental health outcomes. Systematically mapping the existing literature on occupational interventions will provide valuable insights to guide workplace policy development and intervention design.

## Materials and methods

### Study design

A scoping review will be conducted to explore occupational interventions delivered in workplace settings to improve mental health and work participation outcomes. To the best of our knowledge, there are no existing knowledge syntheses that have examined several occupational interventions across the general working population and have evaluated their efficacy in the context of mental health and work-related outcomes. The proposed review will be reported in accordance with the PRISMA-ScR (Preferred Reporting Items for Systematic Reviewers and Meta-Analyses extension for Scoping Reviews) checklist. [21]. This protocol was developed in accordance with the Preferred Reporting Items for Systematic Review and Meta-Analysis Protocols (PRISMA-P) guidelines [22], and the completed checklist can be found in S1 File. The associated review protocol is registered with the Open Science Framework under https://doi.org/10.17605/OSF.IO/EAMNK.

### Search strategy

A literature search will be conducted across five databases to identify relevant articles: Embase, CINAHL, APA PsycINFO, MEDLINE, and Web of Science. The search syntax was developed by two authors (HKCL and SS) and reviewed by the first author (BNK) to ensure accuracy. The search syntax was informed by the Population, Concept, and Context (PCC) framework, as outlined in Table 1. Terms such as “worker”, “employee” (population), “occupational therapy”, “workplace intervention” (concept) and “mental health”, “work participation” (context) are connected through Boolean operators AND and OR. A sample search syntax is provided in Table 2.

**Table 1:** Population, Concept and Context (PCC) framework.

PCC framework	Inclusion criteria	Exclusion criteria
Population	Working adults, 18 years and above, who are currently employed or have the desire to return to work Public safety personnel	<18 years of age, unemployed or have no desire to return-to-work
Concept	Occupational interventions designed to improve workplace mental health and support work participation	Exploring interventions or factors unrelated to workplace mental health, work participation or related outcomes
Context	Workplace settings across industries where mental health and occupational participation outcomes are measured	Editorials, opinion pieces, non-peer-reviewed articles, non-empirical articles, dissertations, pilot studies, reviews
	Articles published in the English language	Non-English language publications
	Articles published from 2005 onwards	Articles published before 2005

**Table 2:** Search syntax for MEDLINE.

Line #	Syntax
**1**	(Worker OR employee OR workforce OR personnel OR staff OR “public safety personnel” OR “public safety officer” OR firefighter OR police OR paramedic OR “first responder” OR “healthcare provider”).ab,kw,ti.
**2**	(“Occupational therapy” OR “occupational intervention” OR “occupational rehabilitation” OR “workplace intervention” OR “employee support” OR “vocational rehabilitation” OR “return to work program” OR “work reintegration”).ab,kw,ti.
**3**	(“Mental health” OR “workplace mental health” OR “emotional health” OR “psychiatric health” OR burnout OR “psychological well-being” OR well-being OR ”occupational stress” OR anxiety OR depression OR engagement OR “work ability” OR “work participation” OR “work engagement” OR “meaningful work” OR “occupational participation”).ab,kw,ti.
**4**	Limit to (English language and yr = ”2005–2025”)

### Study eligibility

Inclusion and exclusion criteria were developed in accordance with the PCC framework (Table 1). Studies will be included if they were peer-reviewed, published in English within the last 20 years. Occupational interventions will be operationalized as interventions that are delivered in occupational contexts and that explicitly target both work participation and workplace mental health among working-aged adults. Studies will be excluded for not having reported on target populations or outcomes, or if the study used have the following study designs: editorials, opinion pieces, non-peer-reviewed articles, non-empirical articles, dissertations, pilot studies, reviews, or grey literature. Studies with the aforementioned designs and grey literature will be excluded to ensure synthesis of peer-reviewed empirical studies with sufficient methodological rigour to allow for meaningful mapping of intervention outcomes. While the exclusion of grey literature may introduce significant limitations, it is beyond the scope of the current review given inconsistencies in indexing and search syntax, which may limit reproducibility. As a result, we call for future research that specifically focuses on grey literature as an extension to the current review.

### Data collection

A comprehensive literature search will be conducted by five independent reviewers (i.e., SS, HKCL, RP, TW, ARS) to compile relevant studies that meet the eligibility criteria. References will be managed using Covidence [23], which is a software tool for collaborative literature reviews. Screening will take place in multiple stages: title and abstract screening, followed by full-text screening. Prior to screening, all reviewers will review the eligibility criteria and discuss example records. During the screening process, criteria may be refined as ambiguities emerge, with previously screened articles being revisited, as necessary. A study will only be included if at least two reviewers agree, and any disagreements between reviewers will be resolved through team meetings and consultation with the first author (BNK). A consensus will be reached before proceeding to the next stage. Inter-rater reliability (Cohen’s kappa) will be evaluated at all stages to support high agreement, accuracy, and quality.

### Data extraction

Data extraction will be conducted among seven independent reviewers (i.e., SS, HKCL, AS, TW, GH, RP, ALFA) following a data charting template in Microsoft Excel [24]. This template will include the following categories: 1) author name(s); 2) publication year; 3) country of origin; 4) study design; 5) demographic characteristics of the population (sample size, age, gender and occupation); 6) outcome conceptualization (definition/conceptualization of mental health and work participation); 7) type of intervention; 8) workplace intervention characteristics (intervention level, duration, delivery mode, provider, intensity/ dose, comparator/control, and follow-up timing); 9) data collection tools (tools used to measure mental health and work participation); and 10) specific outcomes such as impacts on mental health and work participation. The results within each category will inform the identification of a theoretical framework to facilitate thematic organization.

### Data synthesis

Data synthesis will follow the “Arena in work disability prevention model” developed by Loisel and colleagues [25], which situates the individual at the center, with four overarching systems influencing work disability: 1) the personal system and personal coping; 2) the health care system; 3) the workplace system; and, 4) the legislative and insurance systems [25]. Consistent with this model, interventions will be categorized into distinct domains based on their contexts and associations with several work- and health-related outcomes. The categorization process will include a coding procedure, whereby several reviewers will independently classify each intervention into a work disability domain, as described above. Any disagreements will be resolved through team meetings and consultation with the first author (BNK). In the instance that an intervention does not appropriately belong to one of the four domains, re-evaluation of the proposed framework will take place, and potential new domains may be proposed.

## Discussion

The proposed scoping review is designed to identify and categorize interdisciplinary occupational interventions evaluated in workplace studies, map the reported effects from each intervention on work-related and mental health outcomes, highlight evidence gaps, and propose directions for future research. This review will make a meaningful contribution to the literature by synthesizing interdisciplinary occupational interventions across diverse working populations and evaluating their efficacy for two critical outcomes. Understanding the relationships among occupational interventions, work-related outcomes, and mental health outcomes is critical for employees, employers, policy makers, and public health. Furthermore, by avoiding restrictions on working populations and intervention disciplines, we will produce a comprehensive synthesis of relevant and effective occupational interventions for a wide audience. Occupational interventions can reduce symptoms and restore functioning, productivity, and sustained work participation. Systematically mapping intervention types and their impacts across clinical and work-related fields will help clarify which intervention approaches are practical, identify inconsistent or absent evidence, and inform the design of future studies and workplace policies focused on improving mental health and real-world occupational functioning.

Recent reviews highlight that occupational interventions are described and structured in diverse ways, with overlapping labels and definitions, including occupational rehabilitation, work ability interventions, workplace health promotion, and return-to-work programs. The inconsistency in definitions has been identified as a key limiting factor in comparing effects and drawing clear practice recommendations across studies [26]. The proposed review will specifically categorize intervention types, making differences explicit, enabling clearer comparisons, and guiding future workplace intervention designs across diverse workplace settings and populations. The existing literature on mental health outcomes and work outcomes demonstrates that occupational interventions may reduce stress, depression, or burnout, but with inconsistent impacts on attendance, productivity, or work ability [27]. The inconsistency implies that occupational interventions can improve how people feel, without improving their functioning at work. Mapping occupational interventions alongside mental health and work outcomes will help clarify the alignment between interventions and outcomes, inform evidence gaps, and inform interim workplace policies. The Work Disability Prevention framework will be used to explain work disability as the result of interactions between personal factors, workplace conditions, the healthcare system, and broader policy [28]. The Work Disability Prevention framework is widely used to understand how work participation is supported or disrupted, and aligning the proposed review results with it will help clarify which system levels are addressed by current occupational interventions, where important gaps remain, and how occupational strategies could better promote mental health and sustainable work participation.

The proposed breadth for the review is a major strength; specifically, including a wide range of occupational interventions and study designs will capture the diversity of approaches in workplace settings and provide a comprehensive map of available evidence useful to researchers, clinicians, and policymakers. Furthermore, avoiding restrictions on working populations will yield meaningful, evidence-based implications for individuals across diverse workplace settings. The review will necessarily be limited by excluding non-English studies and grey literature, which may reduce the generalizability of our findings and risk omitting relevant interventions or insights that have not entered peer-reviewed English-language literature. This introduces a significant limitation, given that occupational interventions are often targeted to specific working populations and may be funded by local governments. The language restriction limits the generalizability of the current findings and requires that future research implement a protocol to support knowledge synthesis and dissemination across languages. Furthermore, the exclusion of grey literature may introduce publication bias due to the greater likelihood of excluding null or negative findings. The included studies will also vary substantially regarding intervention content, outcome constructs, and measurement methods. The absent standardization across extant studies will complicate cross-study comparisons and quantitative integration analyses. Furthermore, given that the primary aim of the proposed review will be to map the existing literature, a detailed evaluation of intervention efficacy is beyond the scope. As such, future research should aim to expand on the proposed findings to provide more information on the efficacy and feasibility of the interventions to be discussed. Despite the limitation, the proposed scope for the review highlights important patterns and clear priorities for more rigorous, standardized, and inclusive future research.

The review results will inform several important directions for future research. Clarifying which occupational interventions and outcomes appear more robust can inform future research designs and increase alignment between goals, strategies, and outcomes. Mapping gaps across intervention types can also identify where employers, occupational therapists, healthcare providers, and policy makers can work together more closely to support effective occupational interventions focused on mental health outcomes.

## Conclusion

The proposed scoping review is designed to identify and categorize occupational interventions reported in the literature and to map their effects on work- or mental-health-related outcomes. Synthesizing existing evidence will help to clarify how occupational interventions focused on mental health have been defined, implemented, and evaluated across occupational settings. The review results will identify gaps in the literature on intervention types, assessed mental health outcomes, and workplace contexts studied, thereby informing the development, implementation, and evaluation of interventions to improve mental health and work-related well-being.

## Supporting information

S1 File PRISMA-P checklist. (DOCX)
