# Supplementary material for: Occupational interventions and their effects on work-related and mental health outcomes: A scoping review protocol
Source: PLoS One. 2026 Jul 24;21(7):e0354462. doi: 10.1371/journal.pone.0354462 (PMC13399279; doi:10.1371/journal.pone.0354462)
Supplement: Attachment — Submitted filename: Response to Reviewers.docx [file pone.0354462.s003.docx]

**Response to Reviewers**

**Editor Comments**

**Comment #1:** Please ensure that your manuscript meets PLOS ONE’s style requirements, including those for file naming. The PLOS ONE style templates can be found at <https://journals.plos.org/plosone/s/file?id=wjVg/PLOSOne_formatting_sample_main_body.pdf> and <https://journals.plos.org/plosone/s/file?id=ba62/PLOSOne_formatting_sample_title_authors_affiliations.pdf>.

**Response to Comment #1: Thank you for the note. We have revised our file naming and manuscript formatting to abide by PLOS ONE’s style requirements.**

**Comment #2:** Please include captions for your Supporting Information files at the end of your manuscript, and update any in-text citations to match accordingly. Please see our Supporting Information guidelines for more information: <http://journals.plos.org/plosone/s/supporting-information>.

**Response to Comment #2: We have included a caption for our Supporting Information file at the end of our manuscript and matched in-text citations accordingly.**

**Comment #3:** If the review comments include a recommendation to cite specific previously published works, please review and evaluate these publications to determine whether they are relevant and should be cited. There is no requirement to cite these works unless the editor has indicated otherwise.

**Response to Comment #3: This is not applicable to the present reviewer's comments.**

**Comment #4:** Several issues should be addressed before the manuscript can be considered for acceptance. First, the manuscript would benefit from greater conceptual clarity regarding the definition of “occupational interventions”. At present, the term appears to encompass a broad range of approaches (e.g. occupational therapy interventions, workplace interventions, and vocational rehabilitation), and the boundaries between these constructs are not fully delineated. A more precise operational definition would improve the clarity and reproducibility of the review.

**Response to Comment #4: We have revised the Introduction to explicitly state how occupational interventions will be operationalized to improve clarity and reproducibility of the review.**

**Comment #5:** Second, the claim that no prior knowledge syntheses exist in this area should be reconsidered or more carefully substantiated. Given the extensive literature on workplace mental health interventions, including systematic reviews and meta-analyses, it would be important to either temper this statement or explicitly clarify how the present review differs from and advances existing evidence syntheses.

**Response to Comment #5: We have revised these statements to specify how the proposed review will address a meaningful gap in the literature. That is, our review will examine multiple occupational interventions across the general working population and map their efficacy for work- and mental health-related outcomes. In doing so, we will enable a critical comparison of interdisciplinary occupational interventions and their overall efficacy across diverse working populations.**

**Comment #6:** Third, the exclusion of grey literature and certain study types (e.g. pilot studies) warrants further justification. As scoping reviews are typically designed to map the breadth of available evidence, the rationale for these exclusions should be more clearly articulated, including a discussion of how this decision may influence the comprehensiveness of the findings.

**Response to Comment #6: We justified excluding grey literature and other specific study designs to allow for the synthesis of peer-reviewed empirical studies and meaningful comparisons of intervention outcomes. This has been included in the Methods section, and the limitations of this exclusion are in the Discussion section.**

**Comment #7:** Fourth, the Discussion section could be strengthened by more clearly articulating the specific added value of this review. In particular, a more explicit positioning of the anticipated contribute relative to existing literature would enhance the manuscript’s impact.

**Response to Comment #7: We have revised the Discussion section to clearly articulate the specific added value of this review, emphasizing its strengths, including the wide scope and its implications.**

**Comments to the Author**

**1.** Does the manuscript provide a valid rationale for the proposed study, with clearly identified and justified research questions? The research question outlined is expected to address a valid academic problem or topic and contribute to the base of knowledge in the field.

**Reviewer #1:** Yes

**Reviewer #2:** Yes

**2.** Is the protocol technically sound and planned in a manner that will lead to a meaningful outcome and allow testing the stated hypotheses? The manuscript should describe the methods in sufficient detail to prevent undisclosed flexibility in the experimental procedure or analysis pipeline, including sufficient outcome-neutral conditions (e.g. necessary controls, absence of floor or ceiling effects) to test the proposed hypotheses and a statistical power analysis where applicable. As there may be aspects of the methodology and analysis which can only be refined once the work is undertaken, authors should outline potential assumptions and explicitly describe what aspects of the proposed analyses, if any, are exploratory.

**Reviewer #1**: Yes

**Reviewer #2**: Partly.

**3.** Is the methodology feasible and described in sufficient detail to allow the work to be replicable? Descriptions of methods and materials in the protocol should be reported in sufficient detail for another research to reproduce all experiments and analyses. The protocol should describe the appropriate controls, sample size calculations, and replication needed to ensure that the data are robust and reproducible.

**Reviewer #1:** Yes

**Reviewer #2:** No.

**4.** Have the authors described where all data underlying the findings will be made available when the study is complete? The PLOS Data policy requires authors to make all data underlying the findings described in their manuscript fully available without restriction, with rare exception, at the time of publication. The data should be provided as part of the manuscript or its supporting information or deposited to a public repository. For example, in addition to summary statistics, the data points behind means, medians, and variance measures should be available. If there are restrictions on publicly sharing data – e.g. participant privacy or use of data from a third part – those must be specified.

**Reviewer #1:** Yes

**Reviewer #2:** Yes

**5.** Is the manuscript presented in an intelligible fashion and written in standard English? PLOS ONE does not copyedit accepted manuscripts, so the language in submitted articles must be clear, correct, and unambiguous. Any typographical or grammatical errors should be corrected at revision, so please not any specific errors here.

**Reviewer #1:** Yes

**Reviewer #2:** Yes

**6.** Please use the space provided to explain your answers to the questions about an, if applicable, provide comments about issues authors must address before this protocol can be accepted for publication. You may also include additional comments for the author, including concerns about research or publication ethics. You may also provide optional suggestions and comments to authors that they might find helpful in planning their study.

**Reviewer #1**: The research objectives are clearly articulated and address an important gap in the literature regarding workplace mental health and work participation outcomes. The methodological approach is transparent and well-structured, with appropriate database selection, a clearly described screening process, and adherence to PRISMA-ScR and PRISMA-P guidelines. The use of a theoretical framework to guide data synthesis further strengthens the conceptual grounding of the review and enhances its potential relevance for research, practice, and policy. The manuscript presents conceptual ambiguity in its use of the terms “occupational intervention”, “occupation-based interventions”, and “occupational therapy intervention”, which are used interchangeably despite representing distinct constructs. Occupational therapy interventions refer specifically to interventions grounded in occupational therapy theory and delivered within the scope of the profession, whereas “occupational interventions” in workplace literature may broadly refer to job-related or vocational programs delivered by various disciplines. The inclusion of search terms such as “workplace intervention”, “employee support”, and “vocational rehabilitation” further expands the scope beyond occupational therapy. It remains unclear whether the review aims to examine interventions delivered by occupational therapists specifically, or any occupation-focused workplace intervention regardless of disciplinary origin. Clarifying this would substantially enhance methodological coherence and interpretive validity.

**Response to Reviewer #1: Thank you for your comment. Firstly, we have standardized our use of the term “occupational intervention” and removed other terms that were previously being used interchangeably (“occupation-based interventions” and “occupational therapy interventions”). Secondly, we have revised the Introduction to explicitly state how occupational interventions will be operationalized and specified that it will include interventions across several disciplines.**

**Reviewer #2**: This article is very a well-designed proposal, and yet a semi-finished work. Although it “is designed to identify and categorize occupational therapy interventions evaluated in workplace studies, map the reported effects from each intervention on work-related and mental health outcomes, highlight evidence gaps, and propose directions for future research”, it lacks a real final result to accomplish and validate the expected achievements. I would suggest this article to include a process of studying a specific topic for example, such as “the effect of fitness activities in office on depression prevention”, to verify the review protocol is doable, achievable and effective, and people can see the implementation in reality. That is to make the excellent ideas landed.

**Response to Reviewer #2: Thank you for your comment. We feel that including the process of studying a specific topic would be misplaced in this protocol. However, we have referenced several specific examples of occupational interventions that may be included in the full review to offer a glimpse of potential future results.**

**We would like to thank the reviewers for their time and commitment in revising the manuscript. Their comments and suggestions have significantly improved the paper's quality and clarity.**
