# Supplementary material for: Occupational interventions and their effects on work-related and mental health outcomes: A scoping review protocol
Source: PLoS One. 2026 Jul 24;21(7):e0354462. doi: 10.1371/journal.pone.0354462 (PMC13399279; doi:10.1371/journal.pone.0354462)
Supplement: Attachment — Submitted filename: Response to Reviewers_R2.docx [file pone.0354462.s004.docx]

**Response to Reviewers**

**Comments to the Author**

**1.** Does the manuscript provide a valid rationale for the proposed study, with clearly identified and justified research questions? The research question outlined is expected to address a valid academic problem or topic and contribute to the base of knowledge in the field.

**Reviewer #2:** Yes

**2.** Is the protocol technically sound and planned in a manner that will lead to a meaningful outcome and allow testing the stated hypotheses? The manuscript should describe the methods in sufficient detail to prevent undisclosed flexibility in the experimental procedure or analysis pipeline, including sufficient outcome-neutral conditions (e.g. necessary controls, absence of floor or ceiling effects) to test the proposed hypotheses and a statistical power analysis where applicable. As there may be aspects of the methodology and analysis which can only be refined once the work is undertaken, authors should outline potential assumptions and explicitly describe what aspects of the proposed analyses, if any, are exploratory.

**Reviewer #2**: Partly.

**3.** Is the methodology feasible and described in sufficient detail to allow the work to be replicable? Descriptions of methods and materials in the protocol should be reported in sufficient detail for another research to reproduce all experiments and analyses. The protocol should describe the appropriate controls, sample size calculations, and replication needed to ensure that the data are robust and reproducible.

**Reviewer #2:** No.

**4.** Have the authors described where all data underlying the findings will be made available when the study is complete? The PLOS Data policy requires authors to make all data underlying the findings described in their manuscript fully available without restriction, with rare exception, at the time of publication. The data should be provided as part of the manuscript or its supporting information or deposited to a public repository. For example, in addition to summary statistics, the data points behind means, medians, and variance measures should be available. If there are restrictions on publicly sharing data – e.g. participant privacy or use of data from a third part – those must be specified.

**Reviewer #2:** Yes

**5.** Is the manuscript presented in an intelligible fashion and written in standard English? PLOS ONE does not copyedit accepted manuscripts, so the language in submitted articles must be clear, correct, and unambiguous. Any typographical or grammatical errors should be corrected at revision, so please not any specific errors here.

**Reviewer #2:** Yes

**6.** Please use the space provided to explain your answers to the questions about an, if applicable, provide comments about issues authors must address before this protocol can be accepted for publication. You may also include additional comments for the author, including concerns about research or publication ethics. You may also provide optional suggestions and comments to authors that they might find helpful in planning their study.

**Reviewer #2:** This protocol addresses an important topic and has potential value as a broad mapping of workplace occupational interventions affecting mental health and work participation. However, the current methodology requires greater precision regarding:
 definition of occupational interventions,
 screening procedures,
 data extraction variables, and
 planned synthesis methods.

Addressing these issues would substantially improve transparency, reproducibility, and methodological rigor in accordance with PLoS ONE standards.

**Response: Thank you for the recommendations and listing specific sections we can improve on. We have provided definitions for work outcomes used to assess the impact of interventions, revised our screening procedure in the “studies eligibility” section, expanded our data extraction variables, and revised our data synthesis section. Please see individual comments for details.**

**Comment #1:** Conceptual definition of “occupational interventions” remains insufficiently operationalized. A central methodological concern is the broad and potentially ambiguous definition of "occupational interventions." The protocol initially discusses occupational therapy interventions (lines 79–92) but subsequently expands the concept to include workplace mental health interventions, physical activity interventions, vocational rehabilitation programs, and potentially other approaches (lines 93–98). As currently written, the inclusion boundaries remain unclear. For example:
 Would mindfulness interventions be included if they are delivered at work?
 Would organizational-level interventions such as workload redesign qualify?
 Would employee assistance programs (EAPs) be considered occupational interventions?
 Are interventions required to target work participation explicitly?
Without a more precise operational definition, study selection may become inconsistent and difficult to reproduce.

**Response to Comment #1: Thank you for your comment. We have revised the Materials and Methods to provide a more precise operational definition for occupational interventions.**

**Comment #2:** Lack of planned calibration procedures during screening
The protocol states that multiple reviewers will screen studies and disagreements will be resolved through consensus. However, no details are provided regarding reviewer training, pilot screening, calibration exercises, thresholds for acceptable agreement. Merely stating that inter-rater reliability will be evaluated is insufficient. For example, try to describe the statistic to be used (e.g., Cohen's kappa), acceptable agreement thresholds, or calibration procedures before formal screening.

**Response to Comment #2: Thank you for your comment. We have revised the Materials and Methods to provide more clarity on the screening process, including review training, iterative calibration, and inter-rater reliability.**

**Comment #3:** No justification for excluding grey literature. The exclusion of grey literature is justified primarily by methodological rigor (lines 146–147). However, scoping reviews generally seek to comprehensively map available evidence rather than restrict inclusion to highly rigorous studies. Many workplace intervention programs are reported in government reports, employer reports, organizational evaluations, policy documents. Excluding grey literature may substantially limit the comprehensiveness of the evidence map. Either include grey literature or provide stronger methodological justification for exclusion and discuss implications for publication bias.

**Response to Comment #3: We have revised the Materials and Methods to include a stronger methodological justification for exclusion of grey literature. Furthermore, we have revised the Discussion section to discuss the implications of this exclusion for publication bias.**

**Comment #4:** Data extraction framework lacks sufficient detail. The proposed extraction categories are relatively generic. Important variables relevant to workplace intervention research appear absent, including: intervention duration, intervention intensity, delivery mode, intervention provider, organizational level versus individual level intervention, follow-up duration, comparator/control conditions. Without these variables, the planned mapping may not adequately characterize intervention heterogeneity. Expand the extraction framework to include implementation and intervention characteristics.

**Response to Comment #4: Thank you for your suggestion. We have expanded our data extraction framework to include workplace intervention characteristics such as intervention level, duration, delivery mode, provider, intensity/ dose, comparator/control, and follow-up timing. We have also included more information on outcome conceptualization, data collection tools, and specific outcomes for further clarification.**

**Comment #5:** The protocol proposes use of the Arena in Work Disability Prevention Model (lines 170-175), but details remain limited. However, it is unclear how interventions will be mapped to domains, whether coding procedures will be developed, whether multiple reviewers will independently classify interventions, how disagreements will be resolved. Furthermore, not all workplace mental health interventions are explicitly designed around work disability prevention.

**Response to Comment #5: Thank you for your comment. We have revised the Materials and Methods to provide greater clarity on the data synthesis process, including the coding procedure, how disagreements will be resolved, and the protocol for handling an intervention that does not fit the proposed framework.**

**Comment #6:** Potential language bias. Restriction to English-language publications may introduce language bias. The limitation is acknowledged but should also be discussed in relation to workplace intervention literature from non-English-speaking countries.

**Response to Comment #6: Thank you for your comment. We have revised the Discussion section to discuss the limitations of English-language restriction in relation to workplace intervention literature from non-English-speaking countries. We have also called for future research to implement protocols that accommodate such language differences.**

**Comment #7**: Outcome definitions should be standardized. The protocol identifies absenteeism, presenteeism, productivity, work ability, retention, and return-to-work as outcomes. However, no operational definitions are provided. Given substantial variation across studies, predefined outcome definitions would improve consistency during extraction and synthesis.

**Response to Comment #7: Thank you for your comment. We have provided definition of the following terms in the introduction: absenteeism (defined as being absent from work), presenteeism (defined as being present at work by functionally absent), productivity (defined as an employee's ability to complete a given task in an efficient (i.e. completed within a given time) and effective manner), job satisfaction (defined as a person's subjective evaluation regarding their work experiences through feelings of contentment, fulfillment. and happiness) and retention/return to work (defined as resumption of occupational duties/work after a prolonged period of absence).**

**We would like to thank the reviewers for their time and commitment in revising the manuscript. Their comments and suggestions have significantly improved the paper's quality and clarity.**
