# Supplementary material for: Occupational interventions and their effects on work-related and mental health outcomes: A scoping review protocol
Source: PLoS One. 2026 Jul 24;21(7):e0354462. doi: 10.1371/journal.pone.0354462 (PMC13399279; doi:10.1371/journal.pone.0354462)
Supplement: Peer review history

# Peer review: Decision Letter 0

**DOI:** 10.1371/journal.pone.0354462.r001

**Authors:** Andrea Cioffi

23 Mar 2026

PONE-D-26-04522

Occupational Interventions and Their Effects on Work-Related and Mental Health Outcomes: A Scoping Review Protocol

PLOS One

Dear Dr. Nowrouzi-Kia,

Thank you for submitting your manuscript to PLOS ONE. After careful consideration, we feel that it has merit but does not fully meet PLOS ONE’s publication criteria as it currently stands. Therefore, we invite you to submit a revised version of the manuscript that addresses the points raised during the review process.

Please submit your revised manuscript by May 07 2026 11:59PM. If you will need more time than this to complete your revisions, please reply to this message or contact the journal office at plosone@plos.org. When you're ready to submit your revision, log on to https://www.editorialmanager.com/pone/ and select the 'Submissions Needing Revision' folder to locate your manuscript file.

Please include the following items when submitting your revised manuscript:

- A letter that responds to each point raised by the academic editor and reviewer(s). You should upload this letter as a separate file labeled 'Response to Reviewers'.
- A marked-up copy of your manuscript that highlights changes made to the original version. You should upload this as a separate file labeled 'Revised Manuscript with Track Changes'.
- An unmarked version of your revised paper without tracked changes. You should upload this as a separate file labeled 'Manuscript'.

If you would like to make changes to your financial disclosure, please include your updated statement in your cover letter. Guidelines for resubmitting your figure files are available below the reviewer comments at the end of this letter.

If applicable, we recommend that you deposit your laboratory protocols in protocols.io to enhance the reproducibility of your results. Protocols.io assigns your protocol its own identifier (DOI) so that it can be cited independently in the future. For instructions see: https://journals.plos.org/plosone/s/submission-guidelines#loc-laboratory-protocols. Additionally, PLOS ONE offers an option for publishing peer-reviewed Lab Protocol articles, which describe protocols hosted on protocols.io. Read more information on sharing protocols at https://plos.org/protocols?utm_medium=editorial-email&utm_source=authorletters&utm_campaign=protocols.

We look forward to receiving your revised manuscript.

Kind regards,

Andrea Cioffi

Academic Editor

PLOS One

**Journal Requirements:**

When submitting your revision, we need you to address these additional requirements.

1. Please ensure that your manuscript meets PLOS ONE's style requirements, including those for file naming. The PLOS ONE style templates can be found at

https://journals.plos.org/plosone/s/file?id=wjVg/PLOSOne_formatting_sample_main_body.pdf and

https://journals.plos.org/plosone/s/file?id=ba62/PLOSOne_formatting_sample_title_authors_affiliations.pdf

2. Please include captions for your Supporting Information files at the end of your manuscript, and update any in-text citations to match accordingly. Please see our Supporting Information guidelines for more information: http://journals.plos.org/plosone/s/supporting-information.

3. If the reviewer comments include a recommendation to cite specific previously published works, please review and evaluate these publications to determine whether they are relevant and should be cited. There is no requirement to cite these works unless the editor has indicated otherwise.

**Additional Editor Comments:**

Several issues should be addressed before the manuscript can be considered for acceptance.

First, the manuscript would benefit from greater conceptual clarity regarding the definition of “occupational interventions.” At present, the term appears to encompass a broad range of approaches (e.g., occupational therapy interventions, workplace interventions, and vocational rehabilitation), and the boundaries between these constructs are not fully delineated. A more precise operational definition would improve the clarity and reproducibility of the review.

Second, the claim that no prior knowledge syntheses exist in this area should be reconsidered or more carefully substantiated. Given the extensive literature on workplace mental health interventions, including systematic reviews and meta-analyses, it would be important to either temper this statement or explicitly clarify how the present review differs from and advances existing evidence syntheses.

Third, the exclusion of grey literature and certain study types (e.g., pilot studies) warrants further justification. As scoping reviews are typically designed to map the breadth of available evidence, the rationale for these exclusions should be more clearly articulated, including a discussion of how this decision may influence the comprehensiveness of the findings.

Fourth, the Discussion section could be strengthened by more clearly articulating the specific added value of this review. In particular, a more explicit positioning of the anticipated contribution relative to existing literature would enhance the manuscript’s impact.

[Note: HTML markup is below. Please do not edit.]

Reviewers' comments:

Reviewer's Responses to Questions

**Comments to the Author**

1. Does the manuscript provide a valid rationale for the proposed study, with clearly identified and justified research questions?

The research question outlined is expected to address a valid academic problem or topic and contribute to the base of knowledge in the field.

Reviewer #1: Yes

Reviewer #2: Yes

**********

2. Is the protocol technically sound and planned in a manner that will lead to a meaningful outcome and allow testing the stated hypotheses?

The manuscript should describe the methods in sufficient detail to prevent undisclosed flexibility in the experimental procedure or analysis pipeline, including sufficient outcome-neutral conditions (e.g. necessary controls, absence of floor or ceiling effects) to test the proposed hypotheses and a statistical power analysis where applicable. As there may be aspects of the methodology and analysis which can only be refined once the work is undertaken, authors should outline potential assumptions and explicitly describe what aspects of the proposed analyses, if any, are exploratory.

Reviewer #1: Yes

Reviewer #2: Partly

**********

3. Is the methodology feasible and described in sufficient detail to allow the work to be replicable?

Descriptions of methods and materials in the protocol should be reported in sufficient detail for another researcher to reproduce all experiments and analyses. The protocol should describe the appropriate controls, sample size calculations, and replication needed to ensure that the data are robust and reproducible.

Reviewer #1: Yes

Reviewer #2: No

**********

4. Have the authors described where all data underlying the findings will be made available when the study is complete?

The PLOS Data policy requires authors to make all data underlying the findings described in their manuscript fully available without restriction, with rare exception, at the time of publication. The data should be provided as part of the manuscript or its supporting information, or deposited to a public repository. For example, in addition to summary statistics, the data points behind means, medians and variance measures should be available. If there are restrictions on publicly sharing data—e.g. participant privacy or use of data from a third party—those must be specified.

Reviewer #1: Yes

Reviewer #2: Yes

**********

5. Is the manuscript presented in an intelligible fashion and written in standard English?

PLOS ONE does not copyedit accepted manuscripts, so the language in submitted articles must be clear, correct, and unambiguous. Any typographical or grammatical errors should be corrected at revision, so please note any specific errors here.

Reviewer #1: Yes

Reviewer #2: Yes

**********

6. Review Comments to the Author

Please use the space provided to explain your answers to the questions above and, if applicable, provide comments about issues authors must address before this protocol can be accepted for publication. You may also include additional comments for the author, including concerns about research or publication ethics.

You may also provide optional suggestions and comments to authors that they might find helpful in planning their study.

(Please upload your review as an attachment if it exceeds 20,000 characters)

Reviewer #1: The research objectives are clearly articulated and address an important gap in the literature regarding workplace mental health and work participation outcomes. The methodological approach is transparent and well-structured, with appropriate database selection, a clearly described screening process, and adherence to PRISMA-ScR and PRISMA-P guidelines. The use of a theoretical framework to guide data synthesis further strengthens the conceptual grounding of the review and enhances its potential relevance for research, practice, and policy.

The manuscript presents conceptual ambiguity in its use of the terms “occupational intervention,” “occupation-based intervention,” and “occupational therapy intervention,” which are used interchangeably despite representing distinct constructs. Occupational therapy interventions refer specifically to interventions grounded in occupational therapy theory and delivered within the scope of the profession, whereas “occupational interventions” in workplace literature may broadly refer to job-related or vocational programs delivered by various disciplines. The inclusion of search terms such as “workplace intervention,” “employee support,” and “vocational rehabilitation” further expands the scope beyond occupational therapy. It remains unclear whether the review aims to examine interventions delivered by occupational therapists specifically, or any occupation-focused workplace intervention regardless of disciplinary origin. Clarifying this would substantially enhance methodological coherence and interpretive validity.

Reviewer #2: This article is a very well-designed proposal, and yet a semi-finished work. Although it "is designed to identify and categorize occupational therapy interventions evaluated in workplace studies, map the reported effects from each intervention on work-related and mental health outcomes, highlight evidence gaps, and propose directions for future research", it lacks a real final result to accomplish and validate the expected achievements. I would suggest this article to include a process of studying a specific topic for example, such as "the effect of fitness activities in office on depression prevention", to verify the review protocol is doable, achievable and effective, and people can see the implementation in reality. That is to make the excellent ideas landed.

**********

7. PLOS authors have the option to publish the peer review history of their article (what does this mean?). If published, this will include your full peer review and any attached files.

If you choose “no”, your identity will remain anonymous but your review may still be made public.

**Do you want your identity to be public for this peer review?**  For information about this choice, including consent withdrawal, please see our Privacy Policy.

Reviewer #1: No

Reviewer #2: **Yes:** Chuin-Shee Shang

**********

[NOTE: If reviewer comments were submitted as an attachment file, they will be attached to this email and accessible via the submission site. Please log into your account, locate the manuscript record, and check for the action link "View Attachments". If this link does not appear, there are no attachment files.]

To ensure your figures meet our technical requirements, please review our figure guidelines: https://journals.plos.org/plosone/s/figures

You may also use PLOS’s free figure tool, NAAS, to help you prepare publication quality figures: https://journals.plos.org/plosone/s/figures#loc-tools-for-figure-preparation.

NAAS will assess whether your figures meet our technical requirements by comparing each figure against our figure specifications.

# Author comment: Author response to Decision Letter 1

**DOI:** 10.1371/journal.pone.0354462.r002

21 Apr 2026

We would like to thank all reviewers for providing their feedback. We have addressed your comments and made appropriate changes. A more detailed response to each comment is included in a letter attached to the submission.

Attachment Submitted filename: Response to Reviewers.docx

# Peer review: Decision Letter 1

**DOI:** 10.1371/journal.pone.0354462.r003

**Authors:** Andrea Cioffi

8 Jun 2026

PONE-D-26-04522R1

Occupational Interventions and Their Effects on Work-Related and Mental Health Outcomes: A Scoping Review Protocol

PLOS One

Dear Dr. Nowrouzi-Kia,

Thank you for submitting your manuscript to PLOS ONE. After careful consideration, we feel that it has merit but does not fully meet PLOS ONE’s publication criteria as it currently stands. Therefore, we invite you to submit a revised version of the manuscript that addresses the points raised during the review process.

Please submit your revised manuscript by Jul 23 2026 11:59PM. If you will need more time than this to complete your revisions, please reply to this message or contact the journal office at plosone@plos.org. When you're ready to submit your revision, log on to https://www.editorialmanager.com/pone/ and select the 'Submissions Needing Revision' folder to locate your manuscript file.

Please include the following items when submitting your revised manuscript:

- A letter that responds to each point raised by the academic editor and reviewer(s). You should upload this letter as a separate file labeled 'Response to Reviewers'.
- A marked-up copy of your manuscript that highlights changes made to the original version. You should upload this as a separate file labeled 'Revised Manuscript with Track Changes'.
- An unmarked version of your revised paper without tracked changes. You should upload this as a separate file labeled 'Manuscript'.

If you would like to make changes to your financial disclosure, please include your updated statement in your cover letter. Guidelines for resubmitting your figure files are available below the reviewer comments at the end of this letter.

If applicable, we recommend that you deposit your laboratory protocols in protocols.io to enhance the reproducibility of your results. Protocols.io assigns your protocol its own identifier (DOI) so that it can be cited independently in the future. For instructions see: https://journals.plos.org/plosone/s/submission-guidelines#loc-laboratory-protocols. Additionally, PLOS ONE offers an option for publishing peer-reviewed Lab Protocol articles, which describe protocols hosted on protocols.io. Read more information on sharing protocols at https://plos.org/protocols?utm_medium=editorial-email&utm_source=authorletters&utm_campaign=protocols.

As the corresponding author, your ORCID iD is verified in the submission system and will appear in the published article. PLOS supports the use of ORCID, and we encourage all coauthors to register for an ORCID iD and use it as well. Please encourage your coauthors to verify their ORCID iD within the submission system before final acceptance, as unverified ORCID iDs will not appear in the published article. *Only*  the individual author can complete the verification step; PLOS staff *cannot*  verify ORCID iDs on behalf of authors.

We look forward to receiving your revised manuscript.

Kind regards,

Andrea Cioffi

Academic Editor

PLOS One

Journal Requirements:

If the reviewer comments include a recommendation to cite specific previously published works, please review and evaluate these publications to determine whether they are relevant and should be cited. There is no requirement to cite these works unless the editor has indicated otherwise.

**Additional Editor Comments:**

Please, see reviewer's comments.

[Note: HTML markup is below. Please do not edit.]

Reviewers' comments:

Reviewer's Responses to Questions

**Comments to the Author**

1. Does the manuscript provide a valid rationale for the proposed study, with clearly identified and justified research questions?

The research question outlined is expected to address a valid academic problem or topic and contribute to the base of knowledge in the field.

Reviewer #2: Yes

**********

2. Is the protocol technically sound and planned in a manner that will lead to a meaningful outcome and allow testing the stated hypotheses?

The manuscript should describe the methods in sufficient detail to prevent undisclosed flexibility in the experimental procedure or analysis pipeline, including sufficient outcome-neutral conditions (e.g. necessary controls, absence of floor or ceiling effects) to test the proposed hypotheses and a statistical power analysis where applicable. As there may be aspects of the methodology and analysis which can only be refined once the work is undertaken, authors should outline potential assumptions and explicitly describe what aspects of the proposed analyses, if any, are exploratory.

Reviewer #2: Partly

**********

3. Is the methodology feasible and described in sufficient detail to allow the work to be replicable?

Descriptions of methods and materials in the protocol should be reported in sufficient detail for another researcher to reproduce all experiments and analyses. The protocol should describe the appropriate controls, sample size calculations, and replication needed to ensure that the data are robust and reproducible.

Reviewer #2: No

**********

4. Have the authors described where all data underlying the findings will be made available when the study is complete?

The PLOS Data policy requires authors to make all data underlying the findings described in their manuscript fully available without restriction, with rare exception, at the time of publication. The data should be provided as part of the manuscript or its supporting information, or deposited to a public repository. For example, in addition to summary statistics, the data points behind means, medians and variance measures should be available. If there are restrictions on publicly sharing data—e.g. participant privacy or use of data from a third party—those must be specified.

Reviewer #2: Yes

**********

5. Is the manuscript presented in an intelligible fashion and written in standard English?

PLOS ONE does not copyedit accepted manuscripts, so the language in submitted articles must be clear, correct, and unambiguous. Any typographical or grammatical errors should be corrected at revision, so please note any specific errors here.

Reviewer #2: Yes

**********

6. Review Comments to the Author

Please use the space provided to explain your answers to the questions above and, if applicable, provide comments about issues authors must address before this protocol can be accepted for publication. You may also include additional comments for the author, including concerns about research or publication ethics.

You may also provide optional suggestions and comments to authors that they might find helpful in planning their study.

(Please upload your review as an attachment if it exceeds 20,000 characters)

Reviewer #2: This protocol addresses an important topic and has potential value as a broad mapping of workplace occupational interventions affecting mental health and work participation. However, the current methodology requires greater precision regarding:

• definition of occupational interventions,

• screening procedures,

• data extraction variables, and

• planned synthesis methods.

Addressing these issues would substantially improve transparency, reproducibility, and methodological rigor in accordance with PLoS ONE standards.

1. Conceptual definition of “occupational interventions” remains insufficiently operationalized

A central methodological concern is the broad and potentially ambiguous definition of "occupational interventions."

The protocol initially discusses occupational therapy interventions (lines 79–92) but subsequently expands the concept to include workplace mental health interventions, physical activity interventions, vocational rehabilitation programs, and potentially other approaches (lines 93–98). As currently written, the inclusion boundaries remain unclear. For example:

• Would mindfulness interventions be included if they are delivered at work?

• Would organizational-level interventions such as workload redesign qualify?

• Would employee assistance programs (EAPs) be considered occupational interventions?

• Are interventions required to target work participation explicitly?

Without a more precise operational definition, study selection may become inconsistent and difficult to reproduce.

2. Lack of planned calibration procedures during screening

The protocol states that multiple reviewers will screen studies and disagreements will be resolved through consensus.

However, no details are provided regarding reviewer training, pilot screening, calibration exercises, thresholds for acceptable agreement. Merely stating that inter-rater reliability will be evaluated is insufficient. For example, try to describe the statistic to be used (e.g., Cohen's kappa), acceptable agreement thresholds, or calibration procedures before formal screening.

3. No justification for excluding grey literature

The exclusion of grey literature is justified primarily by methodological rigor (lines 146–147). However, scoping reviews generally seek to comprehensively map available evidence rather than restrict inclusion to highly rigorous studies.

Many workplace intervention programs are reported in government reports, employer reports, organizational evaluations, policy documents. Excluding grey literature may substantially limit the comprehensiveness of the evidence map. Either include grey literature or provide stronger methodological justification for exclusion and discuss implications for publication bias.

4. Data extraction framework lacks sufficient detail

The proposed extraction categories are relatively generic. Important variables relevant to workplace intervention research appear absent, including: intervention duration, intervention intensity, delivery mode, intervention provider, organizational level versus individual level intervention, follow-up duration, comparator/control conditions. Without these variables, the planned mapping may not adequately characterize intervention heterogeneity. Expand the extraction framework to include implementation and intervention characteristics.

5. Data synthesis approach requires further elaboration

The protocol proposes use of the Arena in Work Disability Prevention Model (lines 170–175), but details remain limited. However, it is unclear how interventions will be mapped to domains, whether coding procedures will be developed, whether multiple reviewers will independently classify interventions, how disagreements will be resolved. Furthermore, not all workplace mental health interventions are explicitly designed around work disability prevention.

6. Potential language bias

Restriction to English-language publications may introduce language bias. The limitation is acknowledged but should also be discussed in relation to workplace intervention literature from non-English-speaking countries.

7. Outcome definitions should be standardized

The protocol identifies absenteeism, presenteeism, productivity, work ability, retention, and return-to-work as outcomes. However, no operational definitions are provided. Given substantial variation across studies, predefined outcome definitions would improve consistency during extraction and synthesis.

**********

7. PLOS authors have the option to publish the peer review history of their article (what does this mean?). If published, this will include your full peer review and any attached files.

If you choose “no”, your identity will remain anonymous but your review may still be made public.

**Do you want your identity to be public for this peer review?**  For information about this choice, including consent withdrawal, please see our Privacy Policy.

Reviewer #2: **Yes:** Chuin-Shee Shang

**********

[NOTE: If reviewer comments were submitted as an attachment file, they will be attached to this email and accessible via the submission site. Please log into your account, locate the manuscript record, and check for the action link "View Attachments". If this link does not appear, there are no attachment files.]

To ensure your figures meet our technical requirements, please review our figure guidelines: https://journals.plos.org/plosone/s/figures

You may also use PLOS’s free figure tool, NAAS, to help you prepare publication quality figures: https://journals.plos.org/plosone/s/figures#loc-tools-for-figure-preparation.

NAAS will assess whether your figures meet our technical requirements by comparing each figure against our figure specifications.

# Author comment: Author response to Decision Letter 2

**DOI:** 10.1371/journal.pone.0354462.r004

16 Jun 2026

We would like to thank the reviewers for their time and commitment in revising the manuscript. Their comments and suggestions have significantly improved the paper's quality and clarity. Our response to each comment are provided in a separate file.

Attachment Submitted filename: Response to Reviewers_R2.docx

# Peer review: Decision Letter 2

**DOI:** 10.1371/journal.pone.0354462.r005

**Authors:** Andrea Cioffi

9 Jul 2026

Occupational Interventions and Their Effects on Work-Related and Mental Health Outcomes: A Scoping Review Protocol

PONE-D-26-04522R2

Dear Dr. Nowrouzi-Kia,

We’re pleased to inform you that your manuscript has been judged scientifically suitable for publication and will be formally accepted for publication once it meets all outstanding technical requirements.

Within one week, you’ll receive an e-mail detailing the required amendments. When these have been addressed, you’ll receive a formal acceptance letter and your manuscript will be scheduled for publication.

An invoice will be generated when your article is formally accepted. Please note, if your institution has a publishing partnership with PLOS and your article meets the relevant criteria, all or part of your publication costs will be covered. Please make sure your user information is up-to-date by logging into Editorial Manager at Editorial Manager® and clicking the ‘Update My Information' link at the top of the page. For questions related to billing, please contact billing support.

If your institution or institutions have a press office, please notify them about your upcoming paper to help maximize its impact. If they’ll be preparing press materials, please inform our press team as soon as possible -- no later than 48 hours after receiving the formal acceptance. Your manuscript will remain under strict press embargo until 2 pm Eastern Time on the date of publication. For more information, please contact onepress@plos.org.

Kind regards,

Andrea Cioffi

Academic Editor

PLOS One

Additional Editor Comments (optional):

Reviewers' comments:

Reviewer's Responses to Questions

**Comments to the Author**

1. Does the manuscript provide a valid rationale for the proposed study, with clearly identified and justified research questions?

The research question outlined is expected to address a valid academic problem or topic and contribute to the base of knowledge in the field.

Reviewer #2: Yes

**********

2. Is the protocol technically sound and planned in a manner that will lead to a meaningful outcome and allow testing the stated hypotheses?

The manuscript should describe the methods in sufficient detail to prevent undisclosed flexibility in the experimental procedure or analysis pipeline, including sufficient outcome-neutral conditions (e.g. necessary controls, absence of floor or ceiling effects) to test the proposed hypotheses and a statistical power analysis where applicable. As there may be aspects of the methodology and analysis which can only be refined once the work is undertaken, authors should outline potential assumptions and explicitly describe what aspects of the proposed analyses, if any, are exploratory.

Reviewer #2: Yes

**********

3. Is the methodology feasible and described in sufficient detail to allow the work to be replicable?

Descriptions of methods and materials in the protocol should be reported in sufficient detail for another researcher to reproduce all experiments and analyses. The protocol should describe the appropriate controls, sample size calculations, and replication needed to ensure that the data are robust and reproducible.

Reviewer #2: Yes

**********

4. Have the authors described where all data underlying the findings will be made available when the study is complete?

The PLOS Data policy requires authors to make all data underlying the findings described in their manuscript fully available without restriction, with rare exception, at the time of publication. The data should be provided as part of the manuscript or its supporting information, or deposited to a public repository. For example, in addition to summary statistics, the data points behind means, medians and variance measures should be available. If there are restrictions on publicly sharing data—e.g. participant privacy or use of data from a third party—those must be specified.

Reviewer #2: Yes

**********

5. Is the manuscript presented in an intelligible fashion and written in standard English?

PLOS ONE does not copyedit accepted manuscripts, so the language in submitted articles must be clear, correct, and unambiguous. Any typographical or grammatical errors should be corrected at revision, so please note any specific errors here.

Reviewer #2: Yes

**********

6. Review Comments to the Author

Please use the space provided to explain your answers to the questions above and, if applicable, provide comments about issues authors must address before this protocol can be accepted for publication. You may also include additional comments for the author, including concerns about research or publication ethics.

You may also provide optional suggestions and comments to authors that they might find helpful in planning their study.

(Please upload your review as an attachment if it exceeds 20,000 characters)

Reviewer #2: The authors have responded thoughtfully and comprehensively to all of the comments raised during the review process. Their revisions have satisfactorily addressed the concerns, and I am pleased to recommend the manuscript for publication.

**********

7. PLOS authors have the option to publish the peer review history of their article (what does this mean?). If published, this will include your full peer review and any attached files.

If you choose “no”, your identity will remain anonymous but your review may still be made public.

**Do you want your identity to be public for this peer review?**  For information about this choice, including consent withdrawal, please see our Privacy Policy.

Reviewer #2: **Yes:** Chuin-Shee Shang

**********

# Editor report: Acceptance letter

**DOI:** 10.1371/journal.pone.0354462.r006

**Authors:** Andrea Cioffi

PONE-D-26-04522R2

PLOS One

Dear Dr. Nowrouzi-Kia,

I'm pleased to inform you that your manuscript has been deemed suitable for publication in PLOS One. Congratulations! Your manuscript is now being handed over to our production team.

At this stage, our production department will prepare your paper for publication. This includes ensuring the following:

* All references, tables, and figures are properly cited

* All relevant supporting information is included in the manuscript submission,

* There are no issues that prevent the paper from being properly typeset

You will receive further instructions from the production team, including instructions on how to review your proof when it is ready. Please keep in mind that we are working through a large volume of accepted articles, so please give us a few days to review your paper and let you know the next and final steps.

Lastly, if your institution or institutions have a press office, please let them know about your upcoming paper now to help maximize its impact. If they'll be preparing press materials, please inform our press team within the next 48 hours. Your manuscript will remain under strict press embargo until 2 pm Eastern Time on the date of publication. For more information, please contact onepress@plos.org.

You will receive an invoice from PLOS for your publication fee after your manuscript has reached the completed accept phase. If you receive an email requesting payment before acceptance or for any other service, this may be a phishing scheme. Learn how to identify phishing emails and protect your accounts at https://explore.plos.org/phishing.

If we can help with anything else, please email us at customercare@plos.org.

Thank you for submitting your work to PLOS ONE and supporting open access.

Kind regards,

PLOS ONE Editorial Office Staff

on behalf of

Dr. Andrea Cioffi

Academic Editor

PLOS One
